# Adult Antarctic krill proves resilient in a simulated high CO_2_ ocean

**DOI:** 10.1038/s42003-018-0195-3

**Published:** 2018-11-13

**Authors:** Jessica. A. Ericson, Nicole Hellessey, So Kawaguchi, Stephen Nicol, Peter D. Nichols, Nils Hoem, Patti Virtue

**Affiliations:** 1 Institute for Marine and Antarctic Studies, University of Tasmania, 20 Castray Esplanade, Battery Point, TAS 7004 Australia; 2 Antarctic Climate & Ecosystems Cooperative Research Centre, 20 Castray Esplanade, Battery Point, TAS 7004 Australia; 3 CSIRO Oceans and Atmosphere, Castray Esplanade, Battery Point, TAS 7004 Australia; 4 Australian Antarctic Division, 203 Channel Highway, Kingston, TAS 7050 Australia; 5 Aker Biomarine, Oksenøyveien 10, P.O. Box 496, Lysaker, NO-1327 Norway

## Abstract

Antarctic krill (*Euphausia superba*) have a keystone role in the Southern Ocean, as the primary prey of Antarctic predators. Decreases in krill abundance could result in a major ecological regime shift, but there is limited information on how climate change may affect krill. Increasing anthropogenic carbon dioxide (CO_2_) emissions are causing ocean acidification, as absorption of atmospheric CO_2_ in seawater alters ocean chemistry. Ocean acidification increases mortality and negatively affects physiological functioning in some marine invertebrates, and is predicted to occur most rapidly at high latitudes. Here we show that, in the laboratory, adult krill are able to survive, grow, store fat, mature, and maintain respiration rates when exposed to near-future ocean acidification (1000–2000 μatm *p*CO_2_) for one year. Despite differences in seawater *p*CO_2_ incubation conditions, adult krill are able to actively maintain the acid-base balance of their body fluids in near-future *p*CO_2_, which enhances their resilience to ocean acidification.

## Abstract (web summary)

Jessica Ericson et al. examine the viability of Antarctic krill when exposed to near-future levels of ocean acidification for one year. These lab-based simulations of future ocean acidification show that adult krill actively maintain their body acid-base balance, enhancing their resilience.

## Introduction

Increasing anthropogenic carbon dioxide (CO_2_) emissions are causing atmospheric CO_2_ concentrations to rise at a rate unprecedented for millions of years^1^. The global ocean acts as a buffer for rising atmospheric CO_2_ levels, as CO_2_ is sequestered in the surface waters. This absorption of CO_2_ at the air-ocean interface makes seawater more acidic (ocean acidification), due to an increase in the partial pressure of carbon dioxide (*p*CO_2_), hydrogen ions and carbonic acid in seawater^2^. The atmospheric CO_2_ concentration has increased by 120 μatm since the industrial revolution (ca. 1850), causing a 0.1 pH unit drop in ocean surface waters^3,4^. Model projections suggest that if anthropogenic emissions are not reduced this will result in a further decrease of 0.3–0.5 pH units by the year 2100, and 0.77 units by 2300^5,6^.

Ocean acidification has negative effects on some marine organisms, causing decreased mineralisation or dissolution of calcium carbonate shells, decreased or delayed growth, increased mortality and delayed reproduction or abnormalities in offspring^7^. Ocean acidification also causes an increase in *p*CO_2_ (and decrease in pH) in the intra- and extra- cellular spaces of marine organisms, as CO_2_ diffuses across cell membranes^8–10^. The acid–base balance of extracellular fluids must be kept within a certain range for animals to carry out important biochemical functions^11^, prevent metabolic depression and transport oxygen around the body^8^. Despite this range of negative effects, animal responses to acidification are species-specific and a range of positive, negative and neutral responses have been observed in organisms exposed to increased seawater *p*CO_2_ in the laboratory^8,12^. Active crustaceans may be more resilient to ocean acidification than other taxonomic groups, due to their increased ability to regulate extracellular pH (pH_e_) compared with more sessile taxa^9,11^.

*Euphausia superba* (Antarctic krill, hereafter krill) is the primary prey of marine mammals, penguins and seabirds in the Southern Ocean, which makes it a keystone species in this region^13^. Krill are also the target of the region’s largest fishery^14^. They are highly active crustaceans, and their ability to exploit their environment makes them one of the most abundant organisms on Earth^15^.

The Southern Ocean is a major carbon sink^16^ and predictions suggest that ocean acidification will occur most rapidly in this region^17^. Seawater pH in the Southern Ocean varies with season (pH is lower in winter than summer^18^), and *p*CO_2_ is highest at intermediate depths^19,20^. Early life stages of krill (eggs, embryos and larvae) sink to 700–1000 m depths during their development before migrating back to surface waters^21^, and adult krill have been found as deep as 3500 m^22^. Therefore, they may already be exposed to *p*CO_2_ levels up to 550 μatm during their life cycle^20^. Model projections have shown that the Weddell Sea may reach 1000 μatm *p*CO_2_ at the surface_,_ and 2000 μatm *p*CO_2_ at depth, within the next 80 years^19^.

Previous short-term studies indicate that Antarctic krill may be more vulnerable to ocean acidification than crustaceans from lower latitudes. Krill eggs fail to hatch at *p*CO_2_ levels predicted to occur by the year 2300^19,20^, adults increase feeding and nutrient excretion at 750 μatm *p*CO_2_^23^, and krill may not have the behavioural ability to discriminate between low *p*CO_2_ and high *p*CO_2_ seawater^24^.

Understanding how organisms will respond to high CO_2_ requires laboratory experiments that measure a wide range of physiological performance indicators over periods of months or years^9,11,25^. To our knowledge, we conducted the first long-term laboratory study to investigate the effects of ocean acidification on adult Antarctic krill. Adult krill were reared for a 46-week period that encompassed all four seasons (25th January – 12th December 2016). Krill were reared in present day seawater *p*CO_2_ concentrations (400 μatm *p*CO_2,_ the control), a range of seawater *p*CO_2_ levels predicted to occur in their habitat within the next 100–300 years (1000–2000 μatm *p*CO_2_), and an extreme level of 4000 μatm *p*CO_2_. Throughout the 46-week experiment we measured a suite of physiological and biochemical variables, to investigate how future ocean acidification may affect the survival, size (total length), lipid stores (triacylglycerol), reproduction (maturity and female ovarian development), metabolism (respiration rate) and extracellular fluid (haemolymph pH) of krill. We show that these physiological processes in krill are largely unaffected by *p*CO_2_ levels predicted within the next 100–300 years. Adult krill are able to actively maintain their extracellular pH in 400–2000 μatm *p*CO_2_, which enhances their resilience to ocean acidification.

## Results

### Survival

**Fig. 1:**
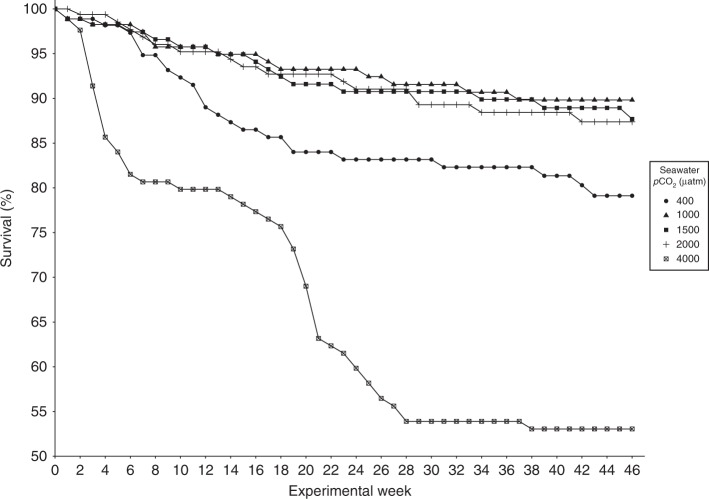
Survival (%) of *Euphausia superba* in each experimental week in 400 (present day control), 1000, 1500, 2000 and 4000 μatm *p*CO_2_ seawater

The survival rate of krill was highest in present day and future *p*CO_2_ seawater (400–2000 μatm) throughout most of the experiment (Fig. 1). The survival rate of krill by week 46 was higher in the 1000–2000 μatm *p*CO_2_ treatments (87–90%) than the control (400 μatm) treatment (79%). Large decreases in survival rate occurred between weeks 3–7 and weeks 19–22 in the extreme *p*CO_2_ (4000 μatm) treatment and plateaued towards the end of the experimental period (weeks 29–46), with 53% of individuals surviving by week 46 (Fig. 1).

### Body length and triacylglycerol content

**Fig. 2:**
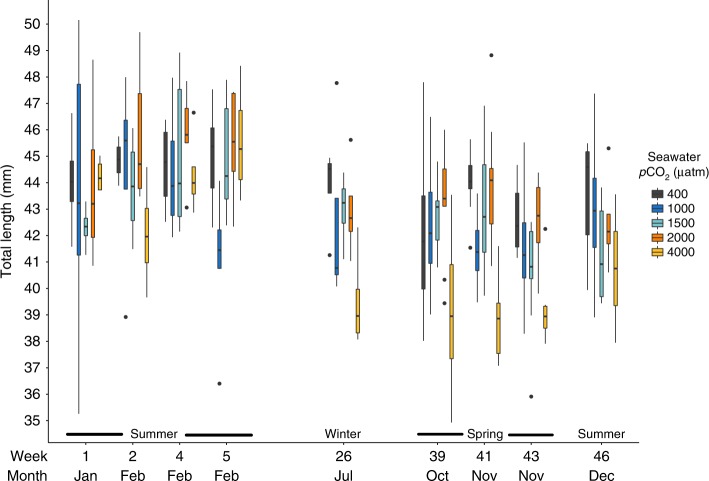
Total length (mm) of *Euphausia superba* in 400, 1000, 1500, 2000 and 4000 μatm *p*CO_2_ seawater in weeks 1, 2, 4, 5, 26, 39, 41, 43 and 46 of the experiment. Box plot elements are centre line, median; box limits, upper and lower quartiles; whiskers extend to the most extreme data point no more than 1.5 times the interquartile range. Shaded black dots denote outlier values that are over 1.5 times the interquartile range. Months and seasons corresponding to the experimental weeks are also provided (*X*-axis). For each *p*CO_2_ by week combination *n* = 2–10. See Supplementary Table 4a for exact sample sizes for each treatment combination

**Fig. 3:**
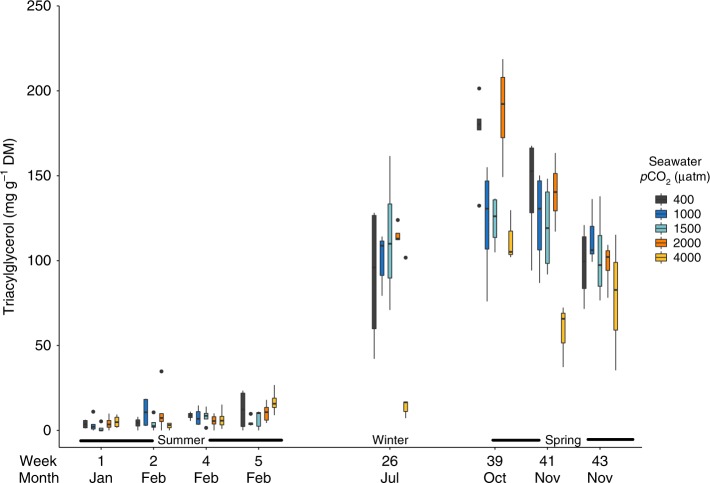
Triacylglycerol (mg g^−1^ dry mass; DM) of *Euphausia superba* in 400, 1000, 1500, 2000 and 4000 μatm *p*CO_2_ seawater in weeks 1, 2, 4, 5, 26, 39, 41 and 43 of the experiment. Box plot elements are centre line, median; box limits, upper and lower quartiles; whiskers extend to the most extreme data point no more than 1.5 times the interquartile range. Shaded black dots denote outlier values that are over 1.5 times the interquartile range. Months and seasons corresponding to the experimental weeks are also provided (*X*-axis). For each *p*CO_2_ by week combination *n* = 3–5. See Supplementary Table 4b for exact sample sizes for each treatment combination

Krill in all treatments maintained their total length and triacylglycerol content (fat stores) during summer (weeks 1, 2, 4 and 5; Fig. 2 and 3), with no differences observed between *p*CO_2_ treatments or weeks for length (Two Way ANOVA, *p*CO_2_*week; df = 12, *F* = 1.12, *p* = 0.359) or triacylglycerol content (Two Way ANOVA, *p*CO_2_*week; df = 12, *F* = 1.14, *p* = 0.341).

By winter (week 26), the median length of krill in all treatments had decreased (Fig. 2). Krill in extreme *p*CO_2_ seawater (4000 μatm) were significantly shorter (Dunnett test, *p* = 0.023) and had stored less fat (Dunnett test, *p* = 0.041) than krill in ambient *p*CO_2_ seawater.

Throughout spring, krill in the 4000 μatm *p*CO_2_ treatment were shorter than krill in 400 μatm *p*CO_2_ (Dunnett tests week 39; *p* *=* 0.094, week 41; *p* *<* 0.001, week 43; *p* *=* 0.005), but no differences were seen between treatments by the following early summer (week 46; One Way ANOVA *p*CO_2_; df = 4, *F* = 0.73, *p* *=* 0.584). Triacylglycerols remained lower in krill in the 4000 μatm *p*CO_2_ treatment compared with krill in ambient *p*CO_2_ throughout early spring (Dunnett tests week 39; *p* = 0.021 and week 41; *p* < 0.001), but all treatments had similar triacylglycerol content by late spring (week 43; One Way ANOVA *p*CO_2_; df = 4, *F* = 1.13, *p* = 0.379).

### Sexual maturation and ovarian development

**Fig. 4:**
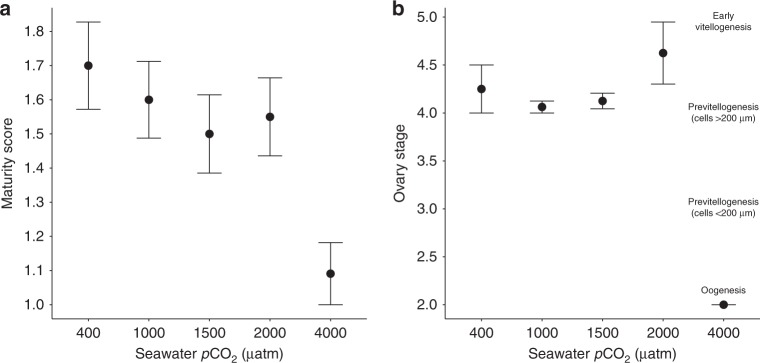
Maturity scores (**a**) and ovary stages (**b**) of *Euphausia superba* in 400, 1000, 1500, 2000 and 4000 μatm *p*CO_2_ seawater. Maturity scores are the average scores (mean ± SE) of krill in each treatment for weeks 39, 41, 43 and 46 combined (*n* = 5 for the 400–2000 μatm *p*CO_2_ treatments and *n* = 3 for the 4000 μatm *p*CO_2_ treatment). Ovary stages are the average ovary stages (mean ± SE) of female krill in week 46 only. The physical ovary stages relating to each stage number are also provided (2 = oogenesis, 3 = previtellogenesis (cell size < 200 µm), 4 = previtellogenesis (cell size > 200 µm), 5 = early vitellogenesis). Higher maturity scores and ovary stages indicate a more advanced reproductive stage. For 400 – 2000 μatm *p*CO_2_ treatments *n* = 8, and for the 4000 μatm *p*CO_2_ treatment *n* = 2

The sexual maturity of krill in the 400–2000 μatm *p*CO_2_ treatments advanced between spring and early summer (weeks 39–46) and all krill reached maturity at similar times (Supplementary Table 1). When maturity scores from weeks 39–46 were combined, overall maturity scores of krill were lowest in the 4000 μatm *p*CO_2_ treatment, suggesting delayed sexual development (Fig. 4a). Krill in 400–2000 μatm *p*CO_2_ had completed ovarian development to the previtellogenesis or early vitellogenesis stages by week 46, but ovarian development in krill in the 4000 μatm *p*CO_2_ treatment was delayed and had not progressed past oogenesis (Fig. 4b).

### Respiration rate

**Fig. 5:**
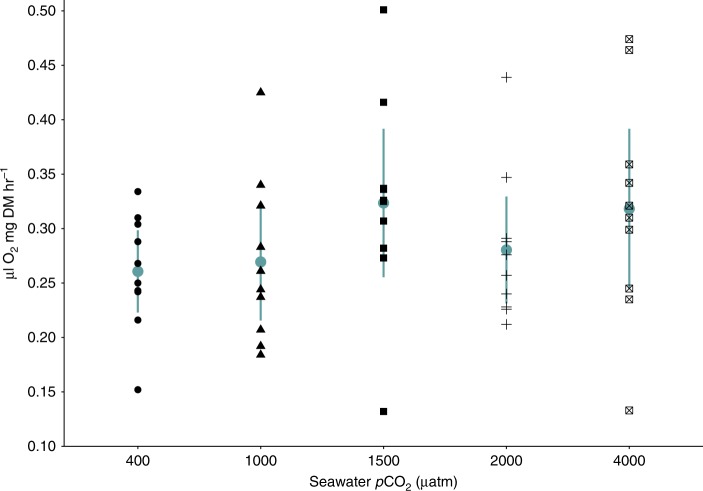
Respiration rates (μL O_2_ mg dry mass h^−1^) of *Euphausia superba* in 400, 1000, 1500, 2000 and 4000 μatm *p*CO_2_ seawater in experimental week 38 (early spring). Each black point denotes the respiration rate of an individual krill (*n* = 10 for each *p*CO_*2*_ treatment). Blue circles denote the mean for each *p*CO_2_ treatment, and error bars represent the 95% confidence intervals for the mean

Respiration rates of krill in early spring (week 38) ranged from 0.13–0.50 μL O_2_ mg DM h^−1^ (Fig. 5) and did not differ between *p*CO_2_ treatments (One Way ANOVA, *p*CO_2_; df = 4, *F* = 1.26, *p* = 0.301). Intraspecific variation in the respiration rates of individual krill increased in elevated *p*CO_2_ treatments (Fig. 5).

### Haemolymph pH

**Fig. 6:**
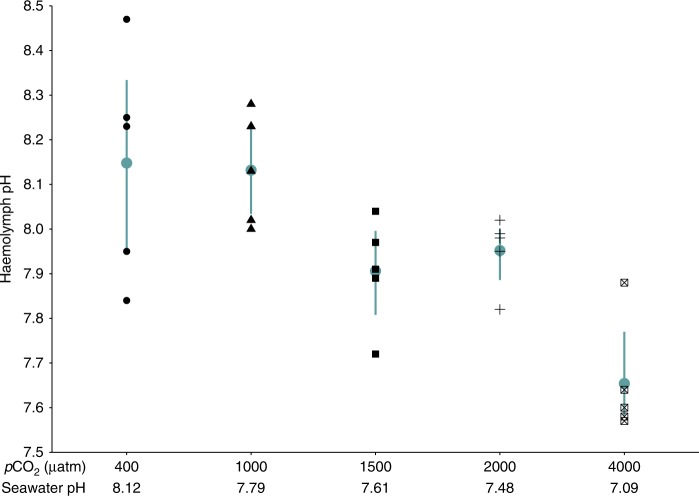
Haemolymph pH of *Euphausia superba* in 400, 1000, 1500, 2000 and 4000 μatm *p*CO_2_ seawater in experimental week 46. Each black point denotes the haemolymph pH of an individual krill (*n* = 5 for each *p*CO_2_ treatment). Blue circles denote the mean for each *p*CO_2_ treatment, and error bars represent the 95% confidence intervals for the mean. Seawater pH values are shown below *p*CO_2_ treatments for ease of interpretation

Haemolymph pH of krill measured in week 46 ranged from pH 7.57–8.47. Haemolymph pH of krill in 1000–2000 μatm *p*CO_2_ treatments did not differ significantly from the control (Dunnett tests; 1000 μatm *p* *=* 1.000, 1500 μatm *p* *=* 0.145, 2000 μatm *p* *=* 0.369) (Fig. 6). The average haemolymph pH of krill in the 4000 μatm *p*CO_2_ treatment was 0.5 units lower than krill in the control treatment (Dunnett test, *p* < 0.001). There was a linear trend of decreasing haemolymph pH with increasing *p*CO_2_ (One Way ANOVA with polynomial contrasts, *p*CO_2_; df = 4, *F* = 11.69, linear *p* < 0.001).

## Discussion

Our experimental results show that the measured physiological processes in adult Antarctic krill were robust to near-future ocean acidification (1000–2000 μatm *p*CO_2_), when elevated *p*CO_2_ was assessed as a single stressor. The survival rate of krill subject to near-future *p*CO_2_ increased by up to 11%, and seasonal patterns of growth, fat storage and reproductive development were comparable to wild krill^26–28^. These physiological processes appeared to be controlled by endogenous rhythms^25,29,30^, and were not affected by near-future *p*CO_2_.

Most studies report a decrease in survival when organisms are exposed to acidification^7^. In contrast, slight increases in euphausiid survival rates have been observed in *Euphausia pacifica* after a 2-month exposure to 1200 μatm *p*CO_2_^31^ and in *Nyctiphanes couchii* after a 35-day exposure to 800 μatm *p*CO_2_ seawater^32^. Euphausiids that are exposed to vertically changing *p*CO_2_ in the water column may use acid-base regulation and short-term metabolic depression (reduced respiration rates) to enhance survival in high *p*CO_2_ conditions^31,33^.

Primary productivity may increase in high *p*CO_2_ seawater, increasing food supply and subsequent survival of herbivores in these experimental treatments^34^. It is unlikely that phytoplankton growth (and therefore food supply) increased in our high *p*CO_2_ tanks, as phytoplankton was grazed by krill within ~2 h. Furthermore, the majority of phytoplankton added to tanks were non-viable cultures that do not photosynthesise. Further targeted studies on krill survival under ocean acidification conditions, and effects of *p*CO_2_ on their food sources, may identify whether krill survival is enhanced in elevated *p*CO_2_ seawater.

In our study, *p*CO_2_ levels between 1000–2000 μatm did not affect the size of adult krill over a whole year and this reflects their ability to moult and grow. Reduced growth rates have been observed in adult crustaceans exposed to high *p*CO_2_ seawater for short-medium term durations (weeks to months)^11^. Elevated *p*CO_2_ did not affect growth rates in the north Atlantic euphausiids *N. couchii*^32^ or *Thysanoessa inermis*^35^ after short-term (5–11 week) exposure, but exposure to levels of 1200 μatm *p*CO_2_ over 2 months slowed growth in *E. pacifica*^31^.

Krill in our study were shorter than wild krill which can grow up to 60 mm in total length^36^. Growth of wild krill is closely related to food quality and quantity, and laboratory reared krill do not grow as large as wild krill^37^. It is impossible to directly replicate the wild diet in controlled conditions, therefore the shorter lengths attained by krill in our study may have been caused by lower food quality in the laboratory. Patterns of seasonal growth seen in wild krill (e.g. winter shrinkage) were, however, observed in our experimental krill, suggesting that the experimental conditions replicated the physiological cycle of wild krill as closely as possible.

The resilience of Antarctic krill, in terms of their maturation and ovarian development to near-future *p*CO_2,_ is comparable to other pelagic crustaceans. Short-term studies (<2 weeks) have generally found that egg production is not affected by moderately increased *p*CO_2_ levels^38,39^, but production rates decrease significantly in crustaceans exposed to extreme *p*CO_2_ levels^40^.

Decreased growth and delayed reproduction are often observed in sessile organisms that cannot maintain their acid-base balance and those that decrease their metabolism when exposed to high *p*CO_2_^7^. This occurs because energy is diverted away from growth and reproduction, and prioritised for acid-base compensation^11^. The ability of active Antarctic krill to maintain their size and mature in 1000–2000 μatm *p*CO_2_ is likely to be directly linked to their ability to maintain acid-base balance and respiration rates at these *p*CO_2_ levels.

An increase in krill metabolic activity has been observed after short term (24 h) exposure to ocean acidification^23^, suggesting that krill may raise their respiration rate on initial exposure to high *p*CO_2_. The increasing variation in krill respiration rates at higher *p*CO_2_ levels suggests that individuals vary in their capacity to respond to CO_2_-induced metabolic stress. This may be due to intraspecific differences in phenotypic plasticity, or genetic predisposition to metabolic resilience in some individuals^41,42^.

The ability of krill to maintain their acid-base balance in elevated *p*CO_2_ seawater may be the key to their successful survival, maturity and growth in a future high CO_2_ world. Haemolymph pH can be increased in hypercapnic conditions via ion transport pumps that pump bicarbonate into the extracellular space^9,43^. These pumps are located in the gill epithelia and consume energy as they actively transport ions in and out of body compartments^8^. Our results suggest that krill in elevated *p*CO_2_ were actively maintaining haemolymph pH, as it remained within the same range for krill in 400–2000 μatm *p*CO_2_. The negligible effects on growth and reproduction in these krill indicate that they were able to actively regulate acid-base balance at low energetic cost. The trend of decreasing haemolymph pH with increasing *p*CO_2_ indicates that although krill in near-future *p*CO_2_ were able to maintain haemolymph pH within the same range as krill in ambient *p*CO_2_, measurements were within the lower range of values for krill in ambient *p*CO_2_. This may have implications for longer term acid-base maintenance. The ability for krill to maintain haemolymph pH beyond one year, and into their spawning season, is unknown. The substantial increase in mortality in extreme *p*CO_2_ (4000 μatm) may have been caused by the inability of those krill to maintain acid-base balance.

Unlike decapods with gills located inside their carapace, Antarctic krill have external gills with a complex structure built for efficient ion and gas exchange^44^. These intricate gills are designed to maximise the amount of O_2_ available to krill during swarming and frequent periods of intense swimming activity^44^. The ability to rapidly exchange O_2_, CO_2_, and ions across their external gills may have assisted krill in maintaining acid-base balance and respiration rates when exposed to near-future acidification. Modification of the respiratory pigment haemocyanin may also assist crustaceans to maintain levels of O_2_ consumption during hypercapnia^11^, however this was not measured in our study.

Krill have evolved a unique range of adaptations to survive the Antarctic winter^29^. Metabolic depression is one such adaptation that is controlled endogenously, cued by the seasonal light cycle^26,45,46^. The physiological responses of krill in our extreme *p*CO_2_ treatment (4000 μatm *p*CO_2_) suggest that this energy-conserving strategy may be less advantageous in high *p*CO_2_ conditions. In winter, as the photoperiod approached 24-h darkness, krill growth and fat deposition in 4000 μatm *p*CO_2_ seawater were reduced compared with krill in ambient *p*CO_2_. In this extreme environment, metabolic depression during winter may have prevented krill from maintaining respiration rates high enough to maintain pH_e_, grow and store fat. These reductions in winter growth and fat storage may have contributed to the subsequent delay in reproductive development^47^.

The energy needed to maintain pH_e_ can be met by consuming more food^48^, and Antarctic krill do increase their feeding rates in elevated *p*CO_2_ seawater^23^. The constant food supply in our experiment may have enabled krill in the 4000 μatm *p*CO_2_ treatment to perform better than if they had received food at seasonally variable concentrations. Importantly, this may have also enabled krill in lower *p*CO_2_ treatments (1000–2000 μatm *p*CO_2_) to maintain haemolymph pH, normal growth, and reproductive development. The relationship between food supply and *p*CO_2_ can affect predator physiology in different ways^49^, and requires further investigation. Metabolic depression, the increasing severity of winter acidification^18^, and regionally variable food concentrations^50^ may increase the vulnerability of krill to near-future ocean acidification during winter.

The prosperity of Antarctic krill in a high CO_2_ world will depend on the ability of adults to produce offspring resilient to ocean acidification. If early life stages cannot survive, this may have catastrophic consequences for krill populations and the Southern Ocean ecosystem. Previous studies indicate that krill eggs and embryos are sensitive to seawater *p*CO_2_ above 1250 μatm^19,20^. These studies used gametes from parents that were maintained in ambient *p*CO_2_ conditions, and gametes were spawned into ambient seawater before being subjected to high *p*CO_2_ conditions. Recent studies have shown that some adult echinoderms and molluscs that acclimate to high *p*CO_2_ conditions are able to produce gametes resilient to high *p*CO_2_^51,52^, and this may allow such species to adapt to ocean acidification over generational time scales^53^. Further studies may establish whether this generational adaptation occurs in krill, which would influence the way that we assess the vulnerability of the early life stages.

Our results suggest that adult Antarctic krill are resilient to ocean acidification, and may not be affected by *p*CO_2_ levels predicted for the next 100–300 years. The overall resilience of Antarctic krill as a species will, however, depend on long-term effects occurring at all life history stages. Endogenous rhythms controlling metabolic rate, combined with food availability in the wild, may influence the vulnerability of krill to high *p*CO_2_ in winter. Negative effects on krill physiology may be seen at near-future *p*CO_2_ levels if effects of acidification are exacerbated by other stressors such as ocean warming. The persistence of krill in the Southern Ocean is vital for the health of the Antarctic ecosystem, and we are only just beginning to understand how this keystone species may respond to climate change.

## Methods

### Experimental conditions

Live krill were collected on the RSV *Aurora Australis* via rectangular mid-water trawl on 22–23 February 2015 (66–03°S, 59–25°E and 66–33°S, 59–35°E). Krill were held in shipboard aquaria using standard maintenance methods^54^ before being transferred to the Australian Antarctic Division’s (AAD) Krill Aquarium in Tasmania (seawater temperature 0.5 °C and pH 8.1). Seawater was supplied to aquarium tanks via a seawater recirculating system^55^.

For ocean acidification experiments, 0.5 °C seawater was supplied from a 70 L header tank and equilibrated with air (control) or CO_2_-enriched air (elevated *p*CO_2_ treatments) before delivery to experimental tanks. The CO_2_-enriched air was monitored using mass flow controllers (Horiba STEC SEC-E-40) and air valves, to regulate flow rates of atmospheric air and pure CO_2_. Five experimental 300 L tanks were maintained at five *p*CO_2_ levels; control 400 μatm *p*CO_2_ (pH 8.1), 1000 μatm *p*CO_2_ (pH 7.8), 1500 μatm *p*CO_2_ (pH 7.6), 2000 μatm *p*CO_2_ (pH 7.4) and 4000 μatm *p*CO_2_ (pH 7.1).

Appropriate tank size and the best possible animal husbandry were high priorities in such a long-term study. As krill are a pelagic species, large sized (300 L) experimental tanks were needed to emulate wild conditions as closely as possible in a laboratory. Our experimental design was limited by the space and resources needed for these large tanks, and our observational units (CO_2_ treatment tanks) could not be replicated. We did not however, observe any visual evidence to suggest that tank effects were confounding our results.

Two hundred krill were randomly assigned to each experimental tank on 25 January 2016, corresponding to a density of 0.6 individuals L^−1^. This density is in the range of 0.5–2 individuals L^−1^ which has been successfully used in previous experiments at the AAD krill aquarium^30,45^. The experiment ran for 46 weeks from the 25 Jan 2016–12 Dec 2016 covering all four seasons. Mortality rates in all *p*CO_2_ treatments (ranging from 0.03–0.2% day^−1^) were much lower than previously reported for Antarctic krill in shipboard aquaria (2% day^−1^)^54^ and in other *p*CO_2_ studies on Pacific krill (0.5% day^−1^)^31^ and northern Atlantic krill (5% day^−1^)^32^.

The *p*CO_2_ levels of the CO_2_-enriched air and seawater were monitored daily using a LI820 CO_2_ gas analyzer and associated computer software (version 2.0.0), and daily pH levels of experimental tanks were measured manually using a pH meter (Mettler Toledo SevenGo Duo Pro). A three-point calibration of the pH meter was undertaken daily using Radiometer Analytical IUPAC Standard buffers of pH 7.000, 7.413 and 9.180. Total alkalinity (A_T_) and dissolved inorganic carbon (DIC) were measured weekly using a Kimoto ATT-05 Total Alkalinity Titrator. Salinity was measured weekly using a Profiline™ Cond 197i Conductivity Meter, WTW. The average total pH (pH_T_), *p*CO_2_, calcite and aragonite saturation (Ω_C_ and Ω_A_) values over the 46 week experiment were calculated in CO_2_SYS^56^ using our measured salinity, temperature, alkalinity and DIC data, and using equilibrium constants of Merhbach, as modified by Dickson and Millero^57^. Average levels of *p*CO_2_ were 8–169 μatm below target levels for the 400–2000 μatm treatments, and 123 μatm above the target level for the 4000 μatm treatment. Seawater temperature and A_T_ were stable in all treatments, while DIC increased with increasing *p*CO_2_. Seawater chemistry in the experimental aquarium is shown in detail in Supplementary Table 2.

Krill were fed 6 days per week with a mixed microalgal diet of the Antarctic flagellate *Pyramimonas gelidicola* at a final concentration of 2 × 10^4^ cells mL^−1^, and Reed Mariculture Inc. (USA) cultures of the diatom *Thalassiosira weissflogii* (8.8 × 10^3^ cells mL^−1^), flagellate *Pavlova lutheri* (4.5 × 10^4^ cells mL^−1^) and flagellate *Isochrysis galbana* (5.5 × 10^4^ cells mL^−^^1^)^30,37^.

Light was controlled in the laboratory to ensure that the photoperiod mimicked the seasonally changing light regime of the Southern Ocean (66°S, 30 m depth). Photoperiod was altered monthly, with a maximum of 100 lux light intensity in February and minimum intensity (24 h darkness) in August (Supplementary Table 3). Light was provided by twin fluorescent tubes and was controlled via standard aquarium procedures^55^.

### Survival

Each *p*CO_2_ treatment was checked daily for mortalities, which were recorded and placed in vials of 10% formalin. Daily mortality data were used to calculate the percentage of krill still surviving at the end of each experimental week in each treatment using the equation:

$${\%}\,{\rm{krill}}\,{\rm{remaining}}\,{\rm{prev}}\,{\rm{week}}-\frac{{{\rm{Num}}\,{\rm{mortalities}}\,{\rm{current}}\,{\rm{week}}}}{{{\rm{Number}}\,{\rm{of}}\,{\rm{krill}}\,{\rm{remaining}}\,{\rm{in}}\,{\rm{tank}}}}\ \times 100$$

where "% krill remaining prev week" is the percentage of krill remaining in the previous week and "Num mortalities current week" is the number of mortalities during the current week. Krill that were sampled for experimental purposes were not counted as mortalities, but were subtracted from the number of krill remaining in the tank each week. This ensured that the remaining number of krill used to calculate survival percentages reflected actual experimental mortality.

### Total length

Krill lengths (mm) were obtained from krill in each *p*CO_2_ treatment in weeks 1, 2, 4, 5, 26, 39, 41, 43 and 46. Sample sizes (*n*) for length measurements for each week and treatment are shown in Supplementary Table 4a. Individuals were sexed using microscopy and the length of each specimen was measured from the tip of the rostrum to the tip of the uropod (measurement Standard Length 1^58^). Length data from frozen krill and live krill were combined.

### Lipid class analysis (triacylglycerols)

Lipid analysis focused on triacylglycerols which are the main storage fat in krill and, therefore, drive overall lipid concentrations and lipid class composition of krill^27^. Krill were sampled for lipid analysis from all *p*CO_2_ treatments in weeks 1, 2, 4, 5, 26, 39, 41 and 43. Individual krill were placed in cryo-tubes and immediately stored in a –80 °C freezer.

Lipid class analysis was carried out on 4-5 krill from *p*CO_2_ treatments 400, 1000, 1500 and 2000 on each sampling week (*n* = 3 for the 4000 μatm tank in weeks 39, 41, and 43 due to increased mortality and lower numbers of krill in that treatment). Sample sizes (*n*) for each week and treatment are shown in Supplementary Table 4b. The wet mass (g), total length (measurement Standard Length 1^58^), and sex for each krill was obtained, and krill were kept frozen during this process to prevent sample degradation. A dry mass (g DM) was obtained later by multiplying the wet mass by 0.2278 to account for the 77.2% water content in the organism^59^. Total lipid extracts of krill specimens were obtained using a modified Bligh and Dyer method^60,61^. Lipid class composition and content were determined using an Iatroscan MK-5 TLC/FID Analyser using standard methods^27^.

### Sexual maturation

The maturity stages of individual krill were identified during weeks 39, 41, 43 and 46 (*n* = 5 for 400–2000 μatm *p*CO_2_ treatments, *n* = 3 for the 4000 μatm *p*CO_2_ treatment). Adult krill undergo sexual regression in winter, so these measurements occurred at the end of the experiment to capture the onset of maturity during late spring/early summer.

The sex and maturity stage of each krill was identified via microscopy (using the staging key in Supplementary Table 5). Each maturity stage was given a maturity score with higher numbers denoting greater maturity (Supplementary Table 5). After staging, individual krill were placed in a cryopreservation tube with 10% formalin.

### Ovarian development

On the final day of the experiment (12 December 2016, Week 46), krill left in each experimental tank were preserved in 10% formalin. These samples were used to determine the ovarian development of eight randomly selected females from each of the 400, 1000, 1500 and 2000 μatm *p*CO_2_ treatments. Only two females remained in the 4000 μatm *p*CO_2_ treatment, therefore only two replicates could be obtained for this tank.

The ovary was dissected out of each organism and a single lobe was placed on a microscope slide with a drop of distilled and deionized water and lightly squashed^62^. Photographs were taken of the ovary section and the lengths of the largest cells (across the longest axis of the cell) were measured using the computer software Image J (https://imagej.nih.gov/ij/). The cell size and photographs were used to determine the maturation stage of krill ovaries using the key in Supplementary Table 6 (modified from Cuzin-Roudy & Amsler^62^). When an ovary was transitioning from one stage to another, a 0.5 value was used (e.g. 4.5). Photographic examples of different ovary stages are shown in Supplementary Figure 1.

### Respiration rate

Respirometry measurements were carried out in experimental week 38. Respirometry vessels (2 L) with pre-fitted O_2_ mini sensors were filled with seawater sourced from the inlet hose of each experimental tank and placed in a 0 °C water bath. Each vessel was connected to O_2_ computer software (version OXY10v3_50TX) via an optic fibre probe.

Ten krill were sampled from each experimental tank (*n* = 50 total) and the total length (measurement Standard Length 1^58^) and wet mass (g) were obtained for each individual. A dry mass (g DM) was obtained by multiplying the wet mass by 0.2278 to account for the 77.2% water content in the organism^59^.

Each krill was then placed into a respirometry vessel completely filled with experimental seawater, with no air spaces in the vessels. Oxygen saturation (%) was logged at 5 min intervals in each respirometry vessel over 22 hrs (9AM–7AM the following day), using the computer software. The software was calibrated at 0 °C and the atmospheric pressure at the time of measurement. After 22-hrs krill were removed from the vessels and returned to their experimental tanks.

Only measurements of O_2_ saturation (%) taken between 12PM–7AM were considered for analysis, to ensure that krill had three hours at the beginning of respiration trials to settle into a normal rhythm of respiration before data was collected. Oxygen saturation (100 %) for seawater at 0 °C and 35.1 salinity units (‰) was converted to O_2_ mL L^−^^1^ using the equation in Fox^63^ to obtain a value of 8.035 mL L^−1^. This was used to convert the O_2_ saturation (%) at each logged time point to millilitres of O_2_ (O_2_ mL) in each 2 L respirometry vessel using the equation:

$${\mathrm{O}}_2\;{\mathrm{ml}}\;{\mathrm{in}}\;{\mathrm{respirometry}}\;{\mathrm{vessel}} = \frac{{\% \ {\mathrm{O}}_2\;{\mathrm{saturation}}}}{{100}} \times \left( {8.035 \times 2} \right)$$

Values for O_2_ mL in each respirometry vessel between 12PM and 7AM were used to create regression equations which were used to compute the O_2_ ml used in each respirometry vessel during this period. This value was divided by the krill dry mass (in mg), converted to µL O_2_ mg DM^−^^1^, then divided by 19 h to obtain the µL O_2_ mg DM hr^−1^.

### Haemolymph pH

The haemolymph pH of five krill from each experimental tank was measured in week 46. Haemolymph pH was measured in situ by inserting a pH microelectrode directly into the pericardial cavity. This ensured that air contact with the haemolymph was minimised, as contact with air may alter the CO_2_ concentration and pH of the body fluids^64^. A Unisense pH Microelectrode (model pH-50, tip diameter 40–60 µm) and Unisense Reference Electrode connected to a Unisense pH/mV Metre and computer software (SensorTrace Logger) were used to complete measurements. The pH microelectrode and reference electrode were calibrated using the SensorTrace software via a three-point calibration using Radiometer Analytical IUPAC Standard pH buffers 7.000, 7.413 and 9.180. The buffers were chilled to the seawater temperature in which haemolymph measurements were conducted (0–0.5 °C). The pH of these buffers at 0 °C was used for calibrations (pH 7.12, 7.53 and 9.46 respectively).

Krill were individually removed from their 300 L tanks and placed under a compound microscope in a refrigerated microscope stage, submerged in seawater from the tank they originated from. The pH of this seawater was measured using the microprobe and reference probe, and a portable pH meter (Mettler Toledo SevenGo Duo Pro) to ensure that the measurements matched to within <0.05 pH units before proceeding.

Live krill were restrained within the microscope stage using acrylic blocks, designed to expose the integument that links the krill carapace to the abdomen. A micromanipulator was used to position the microelectrode relative to the animal. A camera connected to the compound microscope was also used to magnify the krill carapace-abdomen joint and view the real-time image on a computer monitor to ensure the accuracy of microelectrode placement.

The microelectrode was inserted through the integument underneath the carapace and into the pericardial cavity between the thorax and first abdominal segment. The reference probe remained in the seawater surrounding the krill during this process. Some resistance was observed as the microelectrode pierced the integument, causing a slight tear in the body wall as the probe penetrated the integument, ensuring that electrical conductivity was maintained between the reference probe and microelectrode.

The SensorTrace Logger software logged the pH of the haemolymph, and the pH was recorded once the reading had stabilised after ~1 min. The microelectrode was then withdrawn from the abdomen and haemolymph was observed leaking into the surrounding seawater as positive pressure from within the animal pushed it outwards. The krill was removed from the microscope stage and preserved in 10% formalin.

### Statistical analyses

Data were analyzed in the RStudio statistics package (version 0.99.893) using one-way ANOVA with *p*CO_2_ treatment as a factor, or two-way ANOVA with *p*CO_2_ and Week as factors. Dunnett comparisons (carried out using the RStudio multcomp package) were used to identify significant differences between the control treatment (400 μatm *p*CO_2_) and all other factor levels, while Tukey Post-hoc comparisons were used to compare all factor levels with one another. Polynomial contrasts were used to identify linear, quadratic and cubic trends in the data. Type 3 Sums of Squares (SS) were used when data was unbalanced and Type 1 SS were not appropriate. Data were log or square root transformed when assumptions of normality or homogeneity of variances were not met. For all analyses, *α* was set at 0.05 and all tests were two tailed. The RStudio packages ggplot2, plyr and dplyr were used to produce all figures.

## Electronic supplementary material

Supplementary Information

## Data Availability

The datasets generated during and/or analysed during the current study are available from the corresponding author on reasonable request.
