# Supplementary material for: Adult Antarctic krill proves resilient in a simulated high CO2 ocean
Source: Commun Biol. 2018 Nov 13;1:190. doi: 10.1038/s42003-018-0195-3 (PMC6233221; doi:10.1038/s42003-018-0195-3)
Supplement: Supplementary file 1 — Supplementary Information [file 42003_2018_195_MOESM1_ESM.pdf]

Supplementary Information

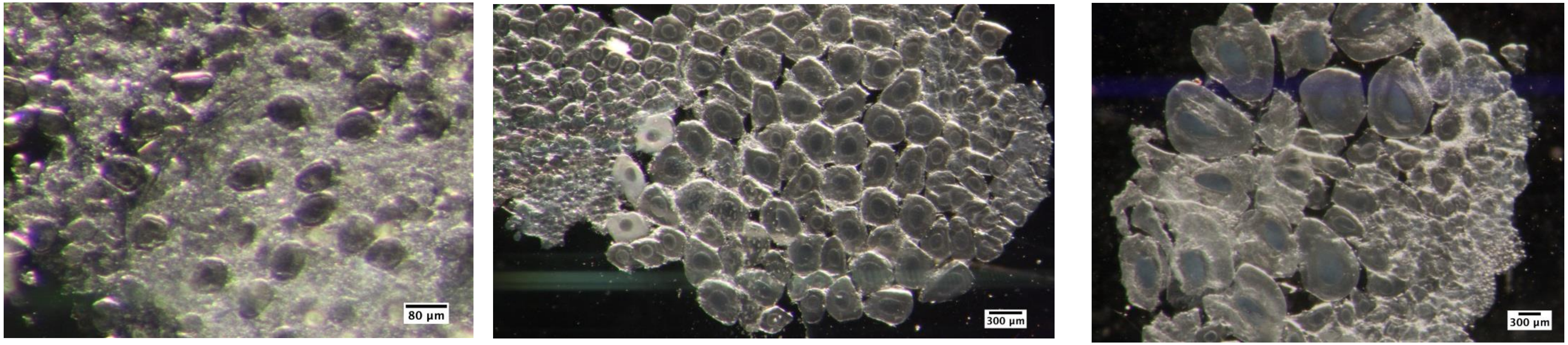

a. Oogenesis **b. Late Previtellogenesis / Early Vitellogenesis** **c. Vitellogenesis**

Supplementary Figure 1. Examples of ovary stages observed in *Euphausia superba*. Ovary stages shown are (a) stage 2, oogenesis (b) stage 4.5, late previtellogenesis/early previtellogenesis and (c) stage 6, vitellogenesis.

Supplementary Table 1. Maturity scores (range and mean) for *Euphausia superba* from each CO_2 treatment, sampled during weeks 39, 41, 43 and 46 (October – December 2016). See Supplementary Table 5 for maturity staging key. For each of the 400, 1000, 1500 and 2000 µatm pCO_2 treatments n = 5, and for the 4000 µatm pCO_2 treatment n = 3.

**Treatment**	**Week 39**		**Week 41**		**Week 43**		**Week 46**	
**(µatm pCO_2)**	**Range**	**Mean**	**Range**	**Mean**	**Range**	**Mean**	**Range**	**Mean**
400	1 – 2	1.6	1 – 2	1.4	1 – 2	1.6	2 – 3	2.2
1000	1 – 2	1.4	1 – 2	1.4	1 – 2	1.4	2	2.0
1500	1 – 2	1.2	1 – 2	1.4	1 – 2	1.6	1 – 2	1.8
2000	1 – 2	1.4	1 – 2	1.4	1 – 2	1.6	1 – 2	1.8
4000	1	1.0	1	1.0	1	1.0	1 – 2	1.5

Supplementary Table 2. Measured pH_{NIST} (mean ± SD), pH_{total} (mean), pCO_2 (mean), seawater temperature (mean ± SD), alkalinity (mean ± SD), dissolved inorganic carbon (mean ± SD), salinity (mean ± SD), and calcite (Ω_C) and aragonite (Ω_A) saturation of seawater in each pCO_2 treatment during the 46-week experimental period (January – December 2016). Values with an asterisk were calculated using CO_2SYS[1].

**Treatment**	**pH_{NIST}** (measured)	**pH_{total}*** (mol/kg SW)	**pCO_2*** (µatm)	**Temp** **(°C)**	**Alkalinity (A_T)** (µmol/kg SW)	**DIC** (µmol/kg SW)	**Salinity** (PSU)	**Ω_C ***	**Ω_A***
400	8.12 (± 0.05)	8.07	392	0.5 (± 0.2)	2456 (± 61)	2320 (± 52)		2.55	1.60
1000	7.79 (± 0.07)	7.73	898	0.5 (± 0.2)	2456 (± 61)	2432 (± 69)		1.25	0.79
1500	7.61 (± 0.06)	7.54	1430	0.5 (± 0.2)	2455 (± 62)	2491 (± 62)	35.3 (± 0.6)	0.82	0.51
2000	7.48 (± 0.07)	7.44	1831	0.5 (± 0.2)	2466 (± 69)	2537 (± 80)		0.65	0.41
4000	7.09 (± 0.08)	7.09	4123	0.5 (± 0.2)	2456 (± 60)	2690 (± 126)		0.30	0.19

Supplementary Table 3. Light regime used during each month of the one-year ocean acidification experiment on adult *Euphausia superba*. Experimental weeks corresponding to each month are also shown in brackets.

Experimental Month	Time lights on	Time lights off	Maximum Lux
January (Week 1)	03:30	20:30	82
February (Weeks 2 – 5)	24 hr daylight	-	100
March (Weeks 6 – 9)	03:30	20:30	82
April (Weeks 10 – 14)	05:30	18:30	64
May (Weeks 15 – 18)	06:00	18:00	45
June (Weeks 19 – 22)	07:30	16:30	27
July (Weeks 23 – 27)	09:30	14:30	9
August (Weeks 28 – 31)	24 hr darkness	-	0
September (Weeks 32 – 36)	09:30	14:30	9
October (Weeks 37 – 40)	07:30	16:30	27
November (Weeks 41 – 44)	06:00	18:00	45
December (Weeks 45 – 46)	05:30	18:30	64

Supplementary Table 4. Sample sizes (n) for *Euphausia superba* (a) total length data and (b) triacylglycerol data for each pCO_2 treatment and experimental week.

pCO_2 Treatment	Week 1	Week 2	Week 4	Week 5	Week 26	Week 39	Week 41	Week 43	Week 46
**(a)** Total Length									
400 µatm	4	4	4	5	4	10	9	9	5
1000 µatm	6	4	6	6	5	9	10	10	5
1500 µatm	4	5	5	7	4	9	9	9	5
2000 µatm	4	5	6	5	4	9	9	9	5
4000 µatm	4	4	5	4	5	6	6	6	2
**(b)** Triacylglycerol									
400 µatm	4	4	4	4	4	5	4	5	n/a
1000 µatm	5	4	4	5	5	4	5	5	n/a
1500 µatm	4	5	4	5	4	4	4	4	n/a
2000 µatm	4	5	5	5	4	4	4	4	n/a
4000 µatm	4	4	5	4	5	3	3	3	n/a

Supplementary Table 5. Maturity stages of subadult and adult *Euphausia superba* and their associated maturity scores. Modified from Seigel[2].

Maturity Stage	Description	Maturity Score
**Male**		
IIA3	subadult male – petasma is two lobed, wing present	1
IIIA	adult male – petasma fully developed	2
**Female**		
IIB	subadult female – developing thelycum present, colour is feeble or absent	1
IIIA	adult female – thelycum fully developed, red in colour, no spermatophores present, body not swollen	2
IIID	gravid female – thorax and first and second abdominal segment swollen by enlarged ovary	3

Supplementary Table 6. Ovary phases and stages of *Euphausia superba* determined from their cell type, cell size (µm) and squash appearance. Modified from tables and keys in Cuzin-Roudy & Amsler[3].

Cell Type	Cell Size (µm)	Squash appearance	Physiological Phase	Ovary Stage
yoc	50 - 150	clear	Oogenesis	2
oc1	150 - 200	translucent	Previtellogenesis	3
	200 - 450	translucent	Previtellogenesis	4
oc2	250 - 450	cloudy and whitish	Early Vitellogenesis	5
oc3	over 450	granular and whitish	Vitellogenesis	6

Supplementary References

1. Pierrot D., Lewis E. & Wallace, D. W. R. MS Excel program developed for CO_2 system calculations. Carbon Dioxide Information Analysis Center, Oak Ridge, 10.3334/CDIAC/otg.CO2SYS_XLS_CDIAC105a (2006).

2. Siegel, V. Introducing Antarctic Krill *Euphausia superba* Dana, 1850 in *Biology and Ecology of Antarctic Krill. Advances in Polar Ecology* (ed. Siegel, V.) 1–19 (Springer International Publishing, 2016).

3. Cuzin-Roudy, J. & Amsler, M. O. Ovarian development and sexual maturity staging in Antarctic krill *Euphausia superba* Dana (Euphausiacea). *J Crustac Biol.* **11**, 236–249 (1991)
